# Fine scale characteristics of catfish aquaculture ponds influencing piscivorous avian species foraging use in the Mississippi Delta

**DOI:** 10.1371/journal.pone.0229402

**Published:** 2020-02-26

**Authors:** Paul C. Burr, Jimmy L. Avery, Garrett M. Street, Bronson K. Strickland, Brian S. Dorr

**Affiliations:** 1 Department of Wildlife, Fisheries, and Aquaculture, Mississippi State University, Mississippi, Mississippi State, United States of America; 2 National Warmwater Aquaculture Center, Mississippi State University, Stoneville, Mississippi, United States of America; 3 Mississippi Field Station, National Wildlife Research Center, Wildlife Services, Animal and Plant Health Inspection Service, United States Department of Agriculture, Mississippi, Mississippi State, United States of America; Universidad Austral de Chile, CHILE

## Abstract

Piscivorous avian species are the main source of catfish depredation at aquaculture facilities in Mississippi, resulting in the economic loss of millions of dollars every year. Most notable of these avian species are the double-crested cormorant (*Phalacrocorax auritus*), great blue heron (*Ardea herodias*), and great egret (*A*. *alba*). Understanding why these species select specific ponds can increase management efficiency directed at avian dispersal and provide insight into their decision making with respect to foraging behavior. We collected species presence data on catfish ponds by flying 35 surveys from October through April of 2015–2017, during which an average of 973 catfish ponds were observed each year. We collected data associated with each pond’s physical surroundings and contents and used occupancy modeling to determine their influence on avian occupancy probability. We also collected data associated with stocking practices and catfish health on a subset of ponds, and constructed resource selection functions to model their influence on avian presence. Pond area was positively related to occupancy probability of each species. Cormorant occupancy increased as pond distance from forest cover and activity centers, such as workshops and offices, increased. Distance to nearest activity center was positively related to egret occupancy, while distance to nearest forested area was negative. Ponds containing diseased catfish had an increased probability of use by both herons and egrets. In general, cormorants and egrets showed greater probability of use on the periphery of pond clusters. The abundance of catfish was positively related to cormorant and heron presence. Specific pond contents and characteristics influenced presence of each avian species in different ways, including fish species cultured, production methods, pond systems, and fish types. Many pond selection relationships were species-specific, illustrating inherent differences in their foraging ecology. Consequently, specific management actions aimed to reduce avian presence will depend on the targeted species.

## Introduction

**Fig 1:**
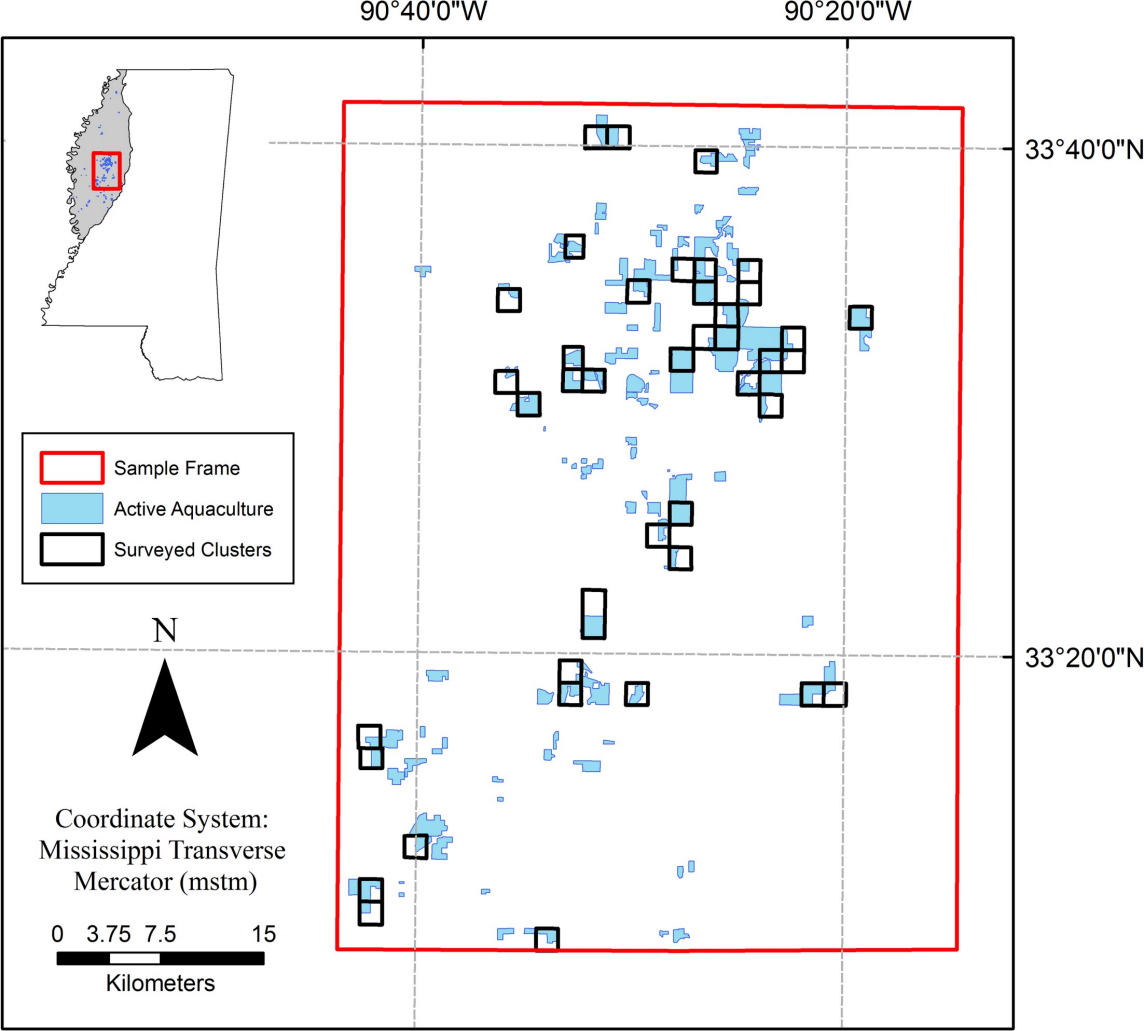
Sample frame established in the Mississippi Delta. Blue polygons represent aquaculture present during the study and black boxes within the sample frame represent aquaculture clusters randomly selected for surveys. Aerial surveys were conducted on these selected clusters to collect presence data of double-crested cormorants (*Phalacrocorax auritus*), great egrets (*Ardea alba*), and great blue herons (*A*. *herodias*). A total of 35 surveys were completed over the months of October through April of three years, 2015–2017.

Commercial production of catfish is the largest aquaculture industry in the United States, with most production (59%) occurring in Mississippi [1]. Sales of domestic catfish products in 2018 were valued at $360 million, of which $207 million came from Mississippi [2]. Most catfish production in Mississippi occurs within an 18,000-km^2^ region located in the northwest portion of the state, known as the Mississippi Delta (Fig 1) [3]. The high concentration of aquaculture facilities found in the Mississippi Delta continually prompts human-wildlife conflict by providing a readily available food source for piscivorous birds inhabiting the region [4–6].

Numerous avian species have been reported to depredate fish at aquaculture facilities throughout North America [7–9]. Double-crested cormorants (*Phalacrocorax auritus;* hereafter, cormorant) are documented as the greatest avian predator of catfish at aquaculture facilities in the southeastern US, followed by great blue herons (*Ardea herodias*; hereafter, herons), and great egrets (*Ardea alba*; hereafter, egrets) [5,8]. Past studies estimate catfish depredation by these species cost producers millions of dollars each year [4,10–13].

Most aquaculture producers have implemented programs to reduce the abundance of foraging birds at their facilities to reduce loss through depredation [8]. These efforts include frightening tactics such as pyrotechnics or gunfire [14], lethal take [15,16], and roost dispersal [17]. These management techniques are large scale, farm-level efforts designed to disperse birds from their facilities. In effect, catfish producers are trying to alter how these birds perceive foraging habitat at aquaculture facilities by increasing the risk associated with catfish ponds in hopes birds will develop an aversion and reduce bird presence over time. Despite these efforts, the foraging opportunities provided by catfish ponds, or the presence of naïve birds, continues to result in human-wildlife conflict through the depredation of catfish. How these avian species select ponds at aquaculture facilities is an area of particular interest for multiple reasons. First, catfish producers can allocate their time and resources to increase the effectiveness of bird dispersal if they have a greater understanding of how birds select for certain ponds over others. Secondly, this will also allow proactive decisions to be made regarding stocking practices or designing future aquaculture facilities to reduce depredation. Lastly, understanding how birds select for certain ponds will add insight into the species’ decision-making behavior with respect to foraging and general habitat use. This is particularly interesting given these species inhabit areas dominated by man-made ponds which are used to augment nutritional requirements. Catfish ponds differ from natural foraging habitat typically used by piscivorous avian species in numerous ways. Catfish ponds are small, uniformly rectangular, spatially clumped, and are accompanied by continuous human presence.

Avian species decision to forage at a group of catfish ponds is analogous to third order habitat selection. Which ponds they forage on within that group of ponds is fourth order habitat selection and our specific area of interest [18]. Therefore, our objective was to evaluate how fine scale factors of individual catfish ponds influence the probability of use by cormorants, herons, and egrets in the Mississippi Delta. We formulated numerous hypotheses regarding individual catfish ponds physical and spatial characteristics influence on how these species select specific ponds for foraging resources over others.

We identified 14 fine scale characteristics to use as variables when examining species use of catfish ponds. These variables can be placed into three principal categories: 1) pond level, 2) surrounding habitat, and 3) conditional (Table 1). Pond level variables include pond size, pond structure, fish type, catfish species, and production method (Table 1). Pond size is the area of a pond, measured in hectares. Catfish ponds in the Delta vary from less than 2 ha up to greater than 9 ha in size, but most are approximately 4 ha [19]. Cormorants are more likely to forage on larger ponds compared to smaller ponds in the region [20]. We predicted similar results for cormorants, herons, and egrets in this study, with the hypothesis that smaller ponds are perceived as riskier or less productive compared to larger ponds.

**Table 1:** Variables of catfish ponds used in occupancy analysis and resource selection functions modeled against the presence of double-crested cormorants, great blue herons, and great egrets on ponds in the Mississippi Delta. Presence data was collected by flying 35 surveys over three consecutive winter seasons (Oct–Apr), 2015–2017.

Variable		Measurement Units
Pond level		
	Pond Size	Hectare
	Pond Structure	Traditional Levee or Split-Pond
	Fish type	Foodfish, Broodfish, or Fingerling
	Catfish species	Channel or Hybrid
	Production Method	Single or Multi-Batch
Surrounding habitat		
	Adjacent pond index	1–4 (categorical)
	Distance: pond to activity centers	Meters
	Distance: pond to all weather roads	Meters
	Distance: pond to forest	Meters
Conditional ^a^		
	Fish abundance	Total fish count
	Date of last stocking	Number of Days
	Presence of disease	Yes or No
	Oxygen level	Low or Normal
	Presence of non-cultured species	Present or Absent

^a^ Variables in which data was only collected during the 2015 winter season from an equal number of randomly selected ponds with, and without target species present. Data for all other variables were collected for each year and individual pond.

Pond structure includes traditional levee ponds or split-pond ponds. Traditional levee ponds are open rectangular ponds stocked with fish that roam freely [21]. Split-pond systems include two ponds that can exchange water, where one pond contains catfish and the other serves as a treatment pond. The treatment pond is a larger, shallow, fishless basin that represents up to 85% of the total water surface area and the fish pond (referred here as split-pond) is stocked with high densities of catfish. The physical separation of fish from pond ecosystem functions such as oxygen control and waste management which occur in the treatment pond can provide greater stocking densities, less maintenance, and increased fish yield [22]. We hypothesized the greater amount of fish found in the split-pond may increase avian predator’s interest and food capture efficiency compared to levee ponds. However, split-ponds also have a smaller surface area which we predicted would be a hindrance.

Fish type categories included broodfish, foodfish, or fingerlings. In Mississippi, approximately 81% of aquaculture acreage is devoted to foodfish, followed by 16% fingerlings, and 3% broodfish [23]. Cormorants and herons typically show higher selection for fingerling ponds [20,24], most likely due to fingerlings being smaller in size, making them easier to capture, handle, and consume. Conversely, larger catfish are more difficult to capture and consume by cormorants, herons, or egrets [25,26]. We therefore predicted broodfish would have the lowest use and fingerlings would have the greatest.

Catfish species included hybrid catfish or channel catfish. The production of hybrid catfish has increased in popularity in recent years [27]. Hybrid catfish are a cross between a female channel catfish (*Ictalurus punctatus*) and a male blue catfish (*I*. *furcatus*). The hybrid is characterized as being superior to the common channel catfish with faster growth rates, better feed conversion, increased disease resistance, and higher tolerance of crowded growth conditions in ponds [28,29]. We predicted hybrids stocked at greater densities would increase avian predation, but hybrids’ resistance to disease and faster growth rates may reduce capture efficiency and therefore reduce overall predation levels.

Production method included multi-batch or single-batch practices. Multi-batch production is a method in which ponds contain more than one age class of fish, and producers selectively harvest fish while continuing to restock the pond. Single-batch production involves ponds containing fish of all the same age, and harvested completely together [19]. Because these avian predators prefer smaller, more easily consumable fish, we predicted the multi-batch system to have more predators present due to the availability of catfish at varying size classes compared to the single-batch system.

Surrounding habitat variables included adjacent pond index and distance from pond to nearest activity center, all-weather roads, and forested areas. The adjacent pond index was a general measure of how isolated ponds were with respect to adjacent ponds. The majority of ponds within our sample were rectangular in shape, generally having four edges. To keep this variable simplistic, we based the index on the presence of neighboring ponds at each edge. For example, if no other ponds were found directly adjacent to the edges of a pond, that ponds’ index would be zero. Alternatively, if a pond was completely surrounded by neighboring ponds the index would be a four. Lower values represent ponds that were more isolated and toward the periphery of clusters, and we predicted the increased isolation would result in greater predator use.

Activity centers were classified as building structures on the farm where personnel are found on a daily basis, including offices, garages, and shops. Activity centers were therefore areas of increased human activity which could reduce bird use of nearby ponds due to perceived risk associated with human presence. All weather roads were characterized as roads that were routinely maintained and regularly accessible. Bird harassment conducted by vehicle is one of the most effective ways to disperse birds. Therefore, pond location relative to these roads may impact species occurrence [14,20]. Finally, we hypothesized distance to forested area may influence avian use of ponds by providing nearby cover and perching sites, or alternatively impact their ability to depart ponds.

Both pond level and surrounding habitat categories included variables that remained constant within a given winter. Conditional category variables, however, could have changed throughout the winter season and their values were therefore conditional upon the timing of each survey. Conditional variables included fish abundance, date of last stocking, presence of disease, oxygen level, and presence of non-cultured species. Average stocking densities (i.e., total fish abundance) has increased in the last ten years [19,21], which may increase avian forage opportunities. For example, cormorants have been reported foraging more on ponds with greater prey densities [30]. We hypothesized that increased abundance of catfish would increase cormorant capture efficiency and predicted the same for herons and egrets.

Date of last stocking (no. of days) was relatable to catfish size, as catfish size during each survey will be dependent on the amount of time since they were stocked. Research suggests herons and egrets select diseased or dead catfish [4,24,26]. Similarly, oxygen levels can impact catfish health and may serve as a proximate cue for piscivorous species [24]. We categorized the variable of disease as present or absent, and oxygen level as normal or low (≤ 3 ppm). We hypothesized reduced health of catfish brought on either by disease or reduced oxygen would reduce their ability to avoid predators, consequently, avian species would select ponds with these conditions. Fish other than primary production species such as shad or carp may be intentionally or unintentionally introduced. The presence of these non-production species can have both positive and negative effects on production [19]. For example, processors may fine producers for having other species in their harvest. However, these species can also potentially serve as an alternate forage resource to birds foraging on catfish ponds [31]. Past studies have found the diets of cormorants wintering in the Delta comprised great amounts of gizzard shad (*Dorosoma cepedianum*) [32]. Similarly, herons and egret diets in the Delta have been reported to contain a large portion of non-catfish species such as *Lepomis* spp. [4]. Therefore, we predicted presence of these non-production fish species would increase foraging selection of avian predators.

## Methods

This work was conducted in a sample frame established in the primary catfish producing area of the Mississippi Delta, predominantly in the counties of Humphreys, Sunflower, and Leflore (Fig 1). The sample frame encompassed 2,772-km^2^ and contained approximately 73% of the total water surface area in production found throughout the entire Delta. To survey avian species foraging on catfish aquaculture ponds within the sample frame we utilized a cluster sampling design [6]. This design consists of aerial surveys flown over aquaculture clusters, which were defined as all USGS land survey sections that contained aquaculture ponds. During aerial surveys target species found on ponds within each cluster were recorded. Ponds were included in the survey if at least 50 percent of its area was within the cluster. We flew surveys over the consecutive winter seasons (October–April) of 2015, 2016, and 2017, coinciding with the peak cormorant movement through Mississippi [6,33]. Cormorants are of particular interest for this work as they are the primary avian predator of catfish aquaculture [9]. Approximately 136 aquaculture clusters were within the sampling frame, but varied between years due to aquaculture facilities and ponds going in and out of production. Of these, we randomly selected 30% (*n* = 41) to be surveyed every year (Fig 1). Thirty percent was chosen for this study to maximize sample size while still being logistically possible to survey within a single day. Our goal was to survey the same clusters each year, however, six clusters in 2015 and one cluster in 2016 ceased production, so we randomly selected replacement clusters for the following year’s survey.

Our aim was to fly two surveys per month from October through April of each year, but was dependent on constraints such as adverse weather or aircraft issues. Each flight was limited to ≤8 hours to ensure counts were completed in one day and to avoid double counting individual birds. Each survey began approximately one hour after sunrise so they could be completed during daylight hours. Surveys were conducted in a fixed-wing aircraft at an altitude of 100–150 m above ground level. The pilot circled over selected clusters and an observer recorded the number of each species present on, or near, each pond within the cluster (i.e., on pond levees). Each target species showed little response to the aircraft, thus movement between ponds within a cluster during surveys was not an issue. Two flight routes were identified in advance, one containing approximately half of the clusters in the north and the other containing the remaining clusters in the south. Each route was structured so the nearest cluster was always surveyed after the proceeding cluster, which served multiple purposes. Firstly, we wanted to increase fuel efficiency to reduce survey costs and time spent refueling the aircraft. Secondly, we wanted to minimize survey time to ensure surveys were completed within a day. Lastly, surveying nearby clusters one after another eliminated the potential for birds to travel between clusters. We randomly selected which route to begin with, and in what direction it was flown for each survey to decrease the probability of surveying the same aquaculture cluster at similar times between surveys.

Following the conclusion of each winter’s surveys, we contacted producers to obtain information of pond structure, fish type, catfish species, and production method of every pond surveyed. Pond size was calculated by manually digitizing ponds in a geographic information system (ArcGIS v 10.2) using high resolution (sub-meter) NAIP (National Agriculture Imagery Program) aerial imagery taken from July to October of 2014, retrieved from the USDA Geospatial Gateway. Distance measurements were similarly calculated in a geographic information system. Data for conditional variables were collected directly from personnel working at the aquaculture facilities within four days after each survey to obtain the most accurate information. Due to logistical constraints, we could not gather conditional variable information for every pond surveyed, so we randomly selected clusters for sampling. Efforts to collect conditional variable data were only made during the 2015 winter season surveys. Only clusters with the target species present were included for selection, and clusters with cormorants, egrets, and herons were selected separately. For each pond within a selected cluster containing the target species, we randomly selected another pond to sample within that cluster that did not have the species present. In the event a selected cluster also contained another target species, we opportunistically collected data for that species as well. This research, including field methods and data collection, was approved under U.S. Department of Agriculture, Wildlife Services, National Wildlife Research Center Quality Assurance protocol, QA-2322, including Institutional Animal Care and Use and attending vet approvals.

### Statistical analysis

#### Occupancy analysis

Our aerial surveys constituted repeated visits of individual ponds over the winter seasons of three years. Our sampling framework allowed use of occupancy modeling, where the probability of a species being present at a site can be estimated while correcting for imperfect detection. Occupancy models allow the ability to model the detection and occupancy processes separately, with the incorporation of related covariates [34]. In these models the definition of sampling sites, replicated sampling occasion, and season are dependent on the application of the model, the situation, and the species [35]. Here, our sampling units are the individual ponds, sampling occasions are the biweekly aerial surveys, and the season was the winter season of each year over the months of October through April. One primary assumption of occupancy modeling is any given site is closed to changes in occupancy between surveys within a given survey season. In our application this meant a pond was assumed to be occupied (or used) throughout the entirety of a winter season if a target species was observed at least once. Although individuals using a pond were not constantly present on that pond throughout the entire sample season, we assumed a pond that was known to be used was likely used by that species continually throughout the winter. All three species are known to be present in the Delta from October through April [36–38], and therefore have the opportunity to use a given pond.

Our primary interested was determining patterns of species occurrence related to pond characteristics rather than processes related to turnover rates associated with dynamic occupancy modeling (i.e., extinction or colonization). We therefore used single season occupancy modeling treating each pond × year combination as a distinct site [39–41]. This places the assumption that the occupancy status of a pond is independent between years, conditional on the covariates modeled. We feel this is a safe assumption given the fine scale in which we selected our sites and the ease at which these species can travel to alternate ponds. Likewise, pond variables rarely changed between years which results in less variability and reduces inference when modeling changes between seasons. We elected to model each species independently rather than using a multispecies approach [34]. Our interest was not in possible interactions these species have or community richness, rather we were interested in how the environmental conditions influenced each species occurrence and predicted occurrence to be species specific.

We used a two-step process to model species occupancy of catfish ponds using package *unmarked* [42] in program R, version 3.5.1 [43]. First, we modeled detection using covariates we hypothesized would affect detection probabilities, while holding occupancy constant. Second, we modeled occupancy using variables corresponding to a priori hypotheses with the best model for detection probability incorporated [44,45]. Because we treated each pond × year as a distinct site, we elected to include year as a categorical variable in all detection and occupancy models constructed, with 2015 set as the reference group.

Abundance of surveyed animals is one of the largest sources of heterogeneity in detectability [46]. The abundances of cormorants, herons, and egrets in the Delta fluctuate throughout the winter season [4,6]. Therefore, we included ordinal date in our detection models, with October 01 = 01. Ordinal date was included up to a third order term to allow detection probabilities to vary over a given season. In the scope of this study, detecting target species on catfish ponds is not necessarily an issue, as the whole pond can be seen from the plane and there was little or no cover for birds to hide. We also found weather conditions, such as cloud cover, did not influence our ability to detect species on ponds. Instead, detection of the target species is related to our survey methods. For instance, we surveyed each pond approximately every two weeks and were typically present at any given pond for a few seconds. If a pond was being used by one of the target species, the probability of us observing them during our brief sampling window was likely low. However, if there were greater abundances of the target species in the area, the chance of observing one of them on any used pond was likely greater. Because each species relative abundance in the Delta was related to ordinal date, we could not include both relative abundance and date in the model. We ran a Pearson’s correlation test between estimated model detection probabilities and total counted species during individual surveys to test the relationship.

After finding the best parameters for the detection model, we then constructed four occupancy models for each species. These models were based on two overarching hypotheses governing pond selection: 1) pond-level variables (i.e., just the pond itself), and/or 2) surrounding habitat variables (i.e., the physical surroundings of a pond). We therefore constructed a model only incorporating year, year and pond level variables, year and surrounding habitat variables, and a global model with all three (Table 1). Occupancy analysis does not allow for missing values of site-level variables which was an issue for the variables of pond structure, fish type, fish species, and production method. For example, broodfish do not necessarily have a production method, and treatment ponds do not contain fish and also lack a production method. In addition, many of these variables were not independent. For example, all fingerling ponds are considered single-batch production and were all in levee ponds, and split-ponds within our sample were all stocked with hybrid foodfish. We therefore combined pond structure, fish type, fish species, and production method into a pond category variable with nine levels that encompass all sampled ponds. These pond categories were broodfish (BR), split-pond hybrid (SH), levee ponds with channel or hybrid catfish in multi-batch production (LCMB, LHMB, respectively), levee ponds with channel or hybrid catfish in single-batch production (LCSB, LHSB, respectively), channel catfish fingerling (CFIN), hybrid catfish fingerling (HFIN), and treatment (TRT) which was set as the reference group.

All continuous variables were standardized to avoid problems with model parameter estimation [47]. We pooled adjacent pond index categories zero and one together due to a small sample size of the zero category, and category four was set as the reference group. We checked for collinearity among predictor variables using variance inflation factors (VIF) calculated from the full model. We used the cut off of VIF > 5 to determine if a variable should be removed [48]. Final detection models, and subsequent occupancy models were chosen based on the smallest value of Akaike information criterion (AIC) [49]. We assessed model fit for the occupancy models using Pearson chi-square [34,50]. This was done by using a parametric bootstrap to generate simulated data based on the global model, which creates detection histories and computes a chi-square value for each iteration [51]. The chi-square produced from the original data was compared to the bootstrapped values. The resulting p-value represents the proportion of bootstrapped values greater than the original value, and a ĉ is calculated from the original chi-square divided by the mean of the bootstrapped values. A ĉ equal to one indicates perfect fit, whereas deviations from one indicate lack of model fit. If lack of fit was evident, quasi-likelihood information criteria is suggested [50]. We ran this test on the global model for every species [49] with 1,000 bootstrap iterations using package *AICcmodavg* [51].

To interpret the influence of continuous variables on species occupancy and detection, along with creating graphical displays, we created multiple linear combinations using the *predict* function in package *unmarked* [42]. This was done by allowing the variable of interest to vary over its range while holding all other variables at their mean. Influence of adjacent pond index, along with yearly estimates of occupancy were calculated in the same way. Because we hypothesized detection would vary over a winter season, we calculated a yearly detection rate for each species by averaging estimates over each year. Finally, for pond category, we averaged all continuous variables by pond category while holding year and adjacent pond index constant. We did this because there were some minor patterns found between pond category and continuous measurement. For example, split-ponds tend to be smaller than levee ponds. We elected not to include an interaction between pond category and any continuous variables due to increasing model complexity associated with the combinations of nine pond categories and multiple continuous variables.

#### Resource selection function

The conditional pond variables could not be included in the occupancy analysis because data were only collected from a subset of aerial-surveyed ponds, and only during the 2015 winter season. We therefore created a resource selection function for each target species by constructing generalized linear mixed models in which we treated individual clusters as random effects using package *lme4* [52] (Table 1). These functions yield values proportional to the probability of use of a resource unit [53]. In this application, we treated resource units as individual catfish ponds and predictor variables were modeled against the binary response of species presence. Our predictor variables included the five conditional variables, as well as fish type to allow for the inclusion of an interaction between fish abundance and fish type. We did this given stocking rates varied considerably between fingerlings and foodfish [23,54]. Treatment ponds and brood ponds were not included for lack of stocking information. All continuous variables were standardized [47], and collinearity was assessed using VIF [48].

## Results

### Occupancy analysis

**Fig 2:**
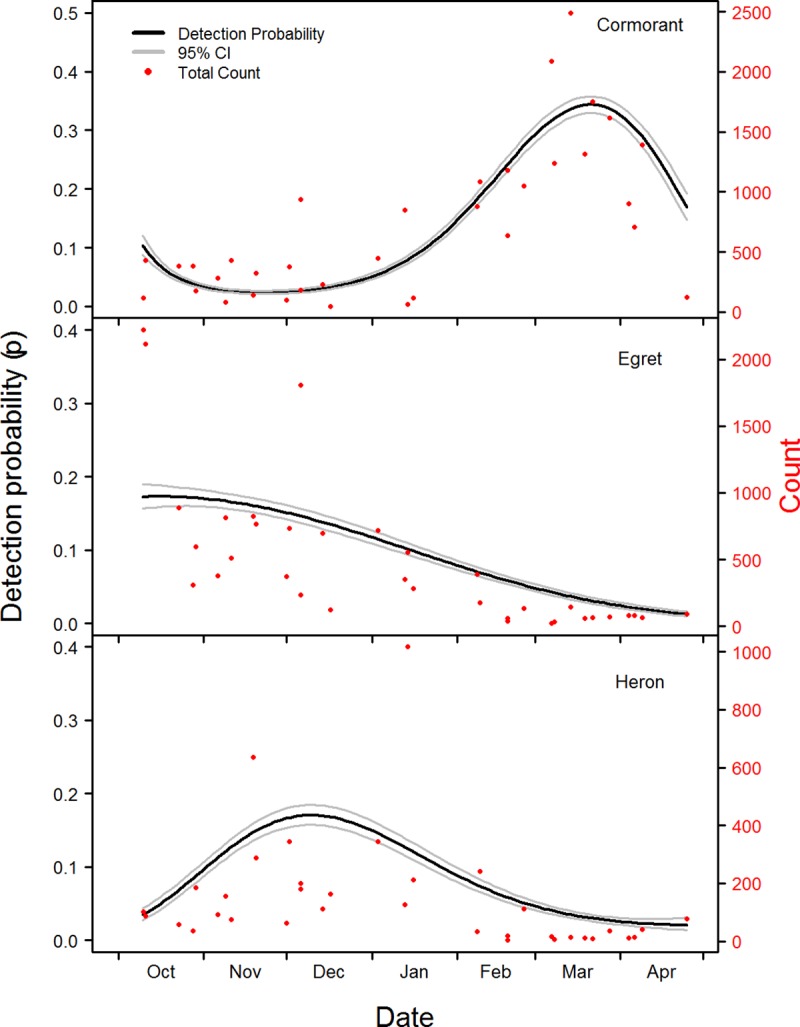
Detection probabilities (+ 95% CI) as a function of date for cormorants, egrets, and herons, estimated from occupancy analysis of catfish pond in the Mississippi Delta. Data used for analysis were collected using aerial surveys of catfish ponds, approximately every two weeks from October–April of 2015, 2016, and 2017. Total count of each survey for each species is displayed in red.

We completed 12 surveys in 2015, 13 surveys in 2016, and 10 in 2017. In total we surveyed 1,187 unique ponds throughout the three years of the study, resulting in 2,883 survey sites included in the occupancy analysis treating each pond × year combination as a unique site (S1 Dataset). Results from the bootstrapped goodness-of-fit estimates using the global models indicated an adequate fit of the data for cormorants (*p* = 0.295, ĉ = 0.970), egrets (*p* = 0.198, ĉ = 1.085), and herons (*p* = 0.163, ĉ = 1.101). Therefore, we applied no adjustment in the model selection processes. There was no evidence of collinearity among any predictor variables (VIFs < 5), so all variables were retained in model construction. With occupancy held constant, parameters for the top detection models included date^3^ for cormorants and herons, and date^2^ for egrets (Tables 2 and 3). Detection varied considerably throughout the winter season, following closely to survey counts (Fig 2). Estimated detection probabilities and total survey count were positively correlated for cormorants (t = 8.52, df = 33, *p* = <0.0001), egrets (t = 4.97, df = 33, *p* = <0.0001), and herons (t = 4.41, df = 33, *p* = 0.0001). Yearly detection estimates averaged over date were consistent for each species, with cormorants having the highest detection followed by egrets and herons (Table 4).

**Table 2:** Detection models (*p*) constructed for cormorants, egrets, and herons with constant occupancy (Ψ) probability. Parameters included in top ranked detection models were included in occupancy model construction. Number of parameters (K), AIC, ΔAIC, and model AIC weight are included. Models were constructed using aerial survey data collected during winters (Oct—Apr) of 2015–2016, 2016–2017 and 2017–2018, of species presence and absence on catfish ponds in the Mississippi Delta. N = 2,883 ponds for each species.

Model ^a^	K	AIC	ΔAIC	AIC Weight
Cormorant				
Ψ(.) *p*(date^3^ + year)	7	16725.3	0.0	1.00
Ψ(.) *p*(date + year)	5	17302.5	577.1	0.0
Ψ(.) *p*(date^2^ + year)	6	17302.8	577.4	0.0
Ψ(.) *p*(year)	4	19368.6	2643.2	0.0
Ψ(.) *p*(.)	2	19401.8	2676.5	0.0
Egret				
Ψ(.) *p*(date^2^ + year)	6	14595.3	0.0	0.72
Ψ(.) *p*(date^3^ + year)	7	14597.2	1.9	0.28
Ψ(.) *p*(date + year)	5	14657.4	62.1	0.0
Ψ(.) *p*(year)	4	15428.5	833.2	0.0
Ψ(.) *p*(.)	2	15501.4	906.1	0.0
Heron				
Ψ(.) *p*(date^3^ + year)	7	11461.4	0.0	1.00
Ψ(.) *p*(date^2^ + year)	6	11518.0	56.5	0.0
Ψ(.) *p*(date + year)	5	11874.3	412.8	0.0
Ψ(.) *p*(year)	4	12107.8	646.3	0.0
Ψ(.) *p*(.)	2	12154.0	692.6	0.0

^a^ Detection covariates: Date: Ordinal date where October 01 of each year is equal to 01. Year: categorical variable of survey year, with 2015 set as the reference

**Table 3:** Model parameter estimates for occupancy and detection for cormorants, egrets, and herons of catfish ponds in the Mississippi Delta. Parameter estimate, standard error, and p-values are shown. Models were constructed using data collected of species presence and absence on catfish ponds in the Mississippi Delta. N = 2,883 ponds for each species.

	Cormorant			Egret			Heron		
Model Variable ^a^	β	SE	*p*	β	SE	*p*	β	SE	*p*
Detection									
Intercept	-2.436			-2.140			-1.976		
Year (2016)	-0.189	0.062	0.002	-0.226	0.080	0.004	-0.177	0.098	0.071
Year (2017)	-0.210	0.063	<0.001	0.222	0.080	0.005	0.435	0.086	<0.001
Date	2.244	0.063	<0.001	-0.794	0.032	<0.001	-1.051	0.075	<0.001
Date^2^	0.398	0.031	<0.001	-0.274	0.035	<0.001	-0.689	0.044	<0.001
Date^3^	-0.780	0.033	<0.001				0.372	0.048	<0.001
Occupancy									
Intercept	-0.802			1.609			-0.320		
Year (2016)	-0.199	0.151	0.187	0.752	0.207	<0.001	-0.355	0.233	0.127
Year (2017)	0.272	0.165	0.098	0.808	0.188	<0.001	0.033	0.205	0.871
*Pond Level*									
LB	0.351	0.246	0.155	-2.009	0.611	0.001	0.398	0.291	0.171
SHSB	1.754	0.261	<0.001	-1.971	0.626	0.001	-0.084	0.320	0.791
LCMB	2.133	0.416	<0.001	1.125	1.911	0.556	1.660	0.486	<0.001
LCSB	2.054	0.419	<0.001	-0.104	1.022	0.919	1.489	0.457	0.001
LHMB	1.477	0.349	<0.001	-3.371	0.641	<0.001	-0.491	0.373	0.118
LHSB	1.561	0.244	<0.001	-2.192	0.580	<0.001	0.537	0.260	0.038
CFIN	0.972	0.262	<0.001	-0.690	0.584	0.238	2.326	0.346	<0.001
HFIN	2.674	0.297	<0.001	-2.458	0.601	<0.001	0.977	0.290	<0.001
Pond Size	0.893	0.093	<0.001	0.600	0.094	<0.001	1.180	0.113	<0.001
*Surrounding Habitat*									
DistActivity	0.357	0.074	<0.001	0.462	0.099	<0.001			
DistRoad	0.081	0.069	0.293	-0.026	0.078	0.738			
DistForest	0.198	0.070	0.004	-0.344	0.077	<0.001			
Adj (1)	0.908	0.375	0.015	1.186	0.479	0.013			
Adj (2)	0.531	0.191	0.005	0.919	0.222	<0.001			
Adj (3)	0.270	0.141	0.056	0.534	0.156	<0.001			

^a^ Detection and Occupancy variables: Year: categorical variable of survey year, with 2015 set as the reference; Date: Ordinal data where October 01 is set to 01 of each year; LB: Levee pond with brood catfish; SHSB: split-pond with hybrid catfish in single-batch production; LCMB: Levee pond with channel catfish in multi-batch production; LCSB: Levee pond with channel catfish in single-batch production; LHMB: Levee pond with hybrid catfish in multi-batch production; LHSB: Levee pond with hybrid catfish in single-batch production; CFIN: channel catfish fingerlings; HFIN: hybrid catfish fingerlings; Treatment ponds were set as the reference group; Pond size: pond area in hectares; DistActivity: distance from the pond to the nearest activity center; DistRoad: distance from the pond to the nearest all weather road; DistForest: distance from the pond to the nearest forested area; Adj: adjacent pond index related to the isolation of individual ponds with category 4 set as the reference group

**Table 4:** Average yearly detection and occupancy rates for cormorants, egrets, and herons on catfish ponds in the Mississippi Delta. Detection varied by date, and the resulting range of detection probabilities are shown. These detection rates represent the probability of observing these species on a pond given the pond is being used, and are only representative of the survey methods described within this study.

Year	Cormorants	Egrets	Herons
	Mean Detection (Range)		
2015	0.16 (0.03–0.37)	0.10 (0.01–0.17)	0.08 (0.02–0.16)
2016	0.14 (0.02–0.33)	0.08 (0.01–0.14)	0.07 (0.02–0.14)
2017	0.14 (0.02–0.33)	0.12 (0.02–0.21)	0.12 (0.03–0.23)
	Mean Occupancy (SE)		
2015	0.74 (0.03)	0.72 (0.07)	0.60 (0.04)
2016	0.70 (0.04)	0.84 (0.04)	0.52 (0.05)
2017	0.79 (0.03)	0.85 (0.04)	0.61 (0.03)

**Fig 3:**
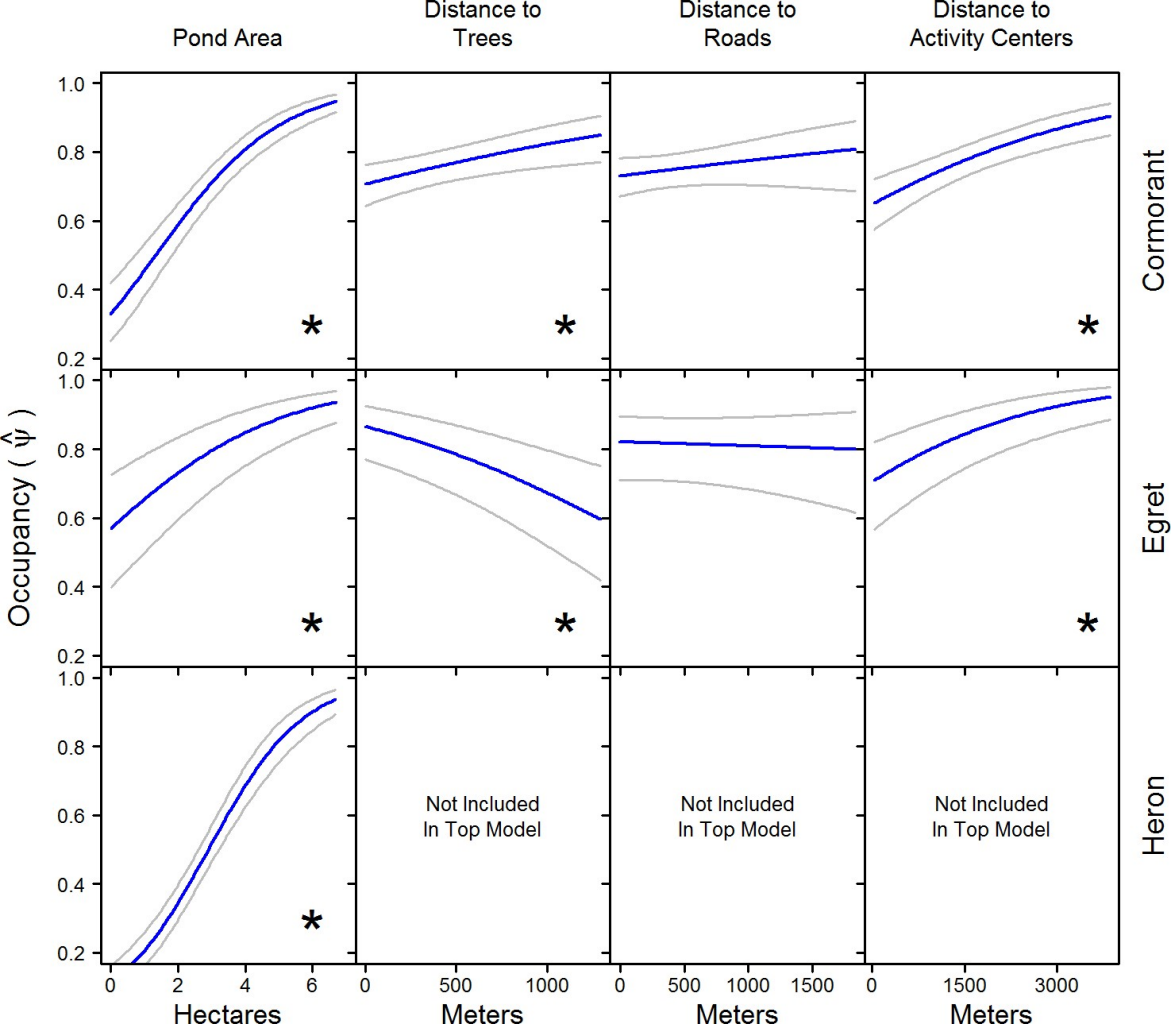
Occupancy probability (+ 95% CI) of catfish ponds in the Mississippi Delta based on continuous variables included in the top ranked models for cormorants, egrets, and herons. Occupancy was estimated from the model by allowing the variable of interest to vary over its range while holding all other variables at their mean. Plots displaying an asterisk (*) indicate a significant result within the model based on an alpha of 0.05.

**Fig 4:**
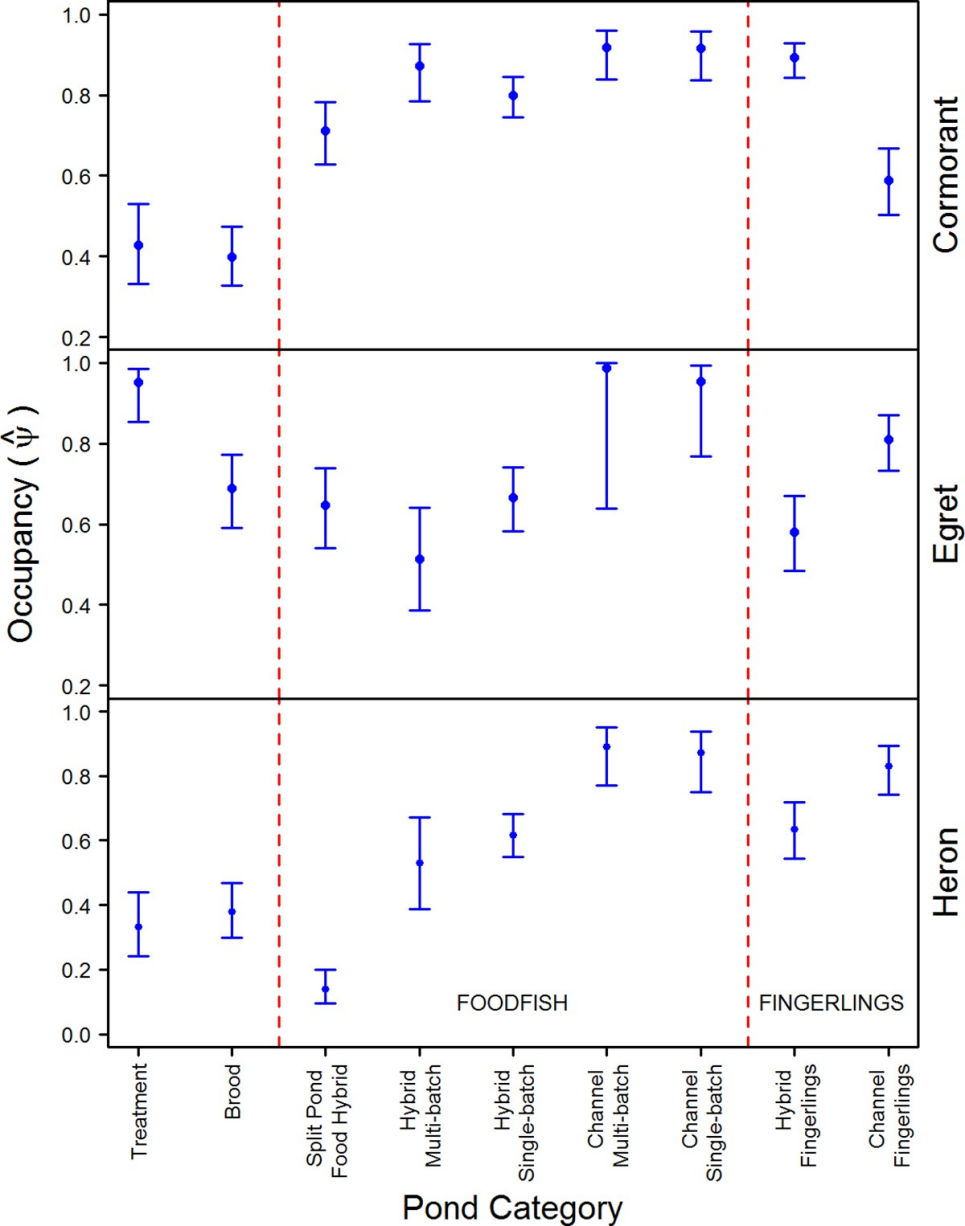
Occupancy probability (+ 95% CI) of catfish ponds in the Mississippi Delta by pond category for cormorants, egrets, and herons. Estimates were made using the top ranked model for each species, while holding all other variables at their mean value based on pond category.

While retaining the best detection parameters, top models for occupancy included pond and surrounding habitat variables for cormorants and egrets, and only pond variables for herons (Tables 3 and 5). Yearly occupancy estimates estimated from the model while holding all other covariates at their mean were consistent among years for each species (Table 4). Pond size had a significant positive influence on occupancy for all three species (Fig 3 and Table 3). Occupancy probabilities based on pond category varied among species (Fig 4 and Table 3). The lowest probabilities for cormorants were on treatment and broodfish ponds. Similar probabilities were estimated between hybrid and channel foodfish ponds, with hybrid single-batch showing a slightly lesser probability and hybrid foodfish in split-pond systems having the lowest probability. Occupancy on hybrid fingerlings was comparable to foodfish categories, but occupancy on channel fingerlings was only greater than that of treatment and broodfish ponds. Occupancy probabilities for egrets were more variable between pond categories (Fig 4). Treatment ponds and channel foodfish ponds had the highest occupancy probabilities, while brood, split-pond, and hybrid foodfish ponds were similar. Egret occupancy on hybrid fingerlings was less than channel fingerlings. Heron occupancy was lowest on split-ponds, followed by treatment and brood ponds. Heron occupancy was similar between single-batch and multi-batch for each catfish species, but overall channel catfish had higher occupancy probabilities than hybrid. Similar to egrets, herons had higher occupancy on channel fingerlings compared to hybrid fingerlings (Fig 4).

**Table 5:** Occupancy models (Ψ) constructed for cormorants, egrets, and herons using parameters from the top ranked model for detection (*p*). Number of parameters (K), AIC, ΔAIC, and models AIC weight are included. Models were constructed using data collected of species presence and absence on catfish ponds in the Mississippi Delta. N = 2,883 ponds for each species.

Model ^a^	K	AIC	ΔAIC	AIC Weight
Cormorant				
Ψ(pond + surroundings + year) *p*(date^3^ + year)	24	16290.5	0	1.0
Ψ(pond + year) *p*(date^3^ + year)	18	16326.6	36.0	0.0
Ψ(surroundings + year) *p*(date^3^ + year)	15	16688.4	397.8	0.0
Ψ(year) *p*(date^3^ + year)	9	16723.6	433.1	0.0
Ψ(.) *p*(.)	2	19401.8	3111.3	0.0
Egret				
Ψ(pond + surroundings + year) *p*(date^2^ + year)	23	14321.8	0	1.0
Ψ(pond + year) *p*(date^2^ + year)	17	14409.9	88.0	0.0
Ψ(surroundings + year) *p*(date^2^ + year)	14	14510.8	189.0	0.0
Ψ(year) *p*(date^2^ + year)	8	14585.1	263.3	0.0
Ψ(.) *p*(.)	2	15501.4	1179.6	0.0
Heron				
Ψ(pond + year) *p*(date^3^ + year)	18	11048.3	0	0.88
Ψ(pond + surroundings + year) *p*(date^3^ + year)	24	11052.3	4.0	0.12
Ψ(surroundings + year) *p*(date^3^ + year)	15	11393.5	345.2	0.0
Ψ(year) *p*(date^3^ + year)	9	11460.6	412.3	0.0
Ψ(.) *p*(.)	2	12154.0	1105.7	0.0

^a^ Variable description: Year: categorical variable of survey year, with 2015 set as the reference; Date: Ordinal data where October 01 is set to 01 of each year; pond: Group of variables related to individual catfish ponds (Table 1); Surroundings: Group of variables related to the physical surroundings of catfish ponds (Table 1)

**Fig 5:**
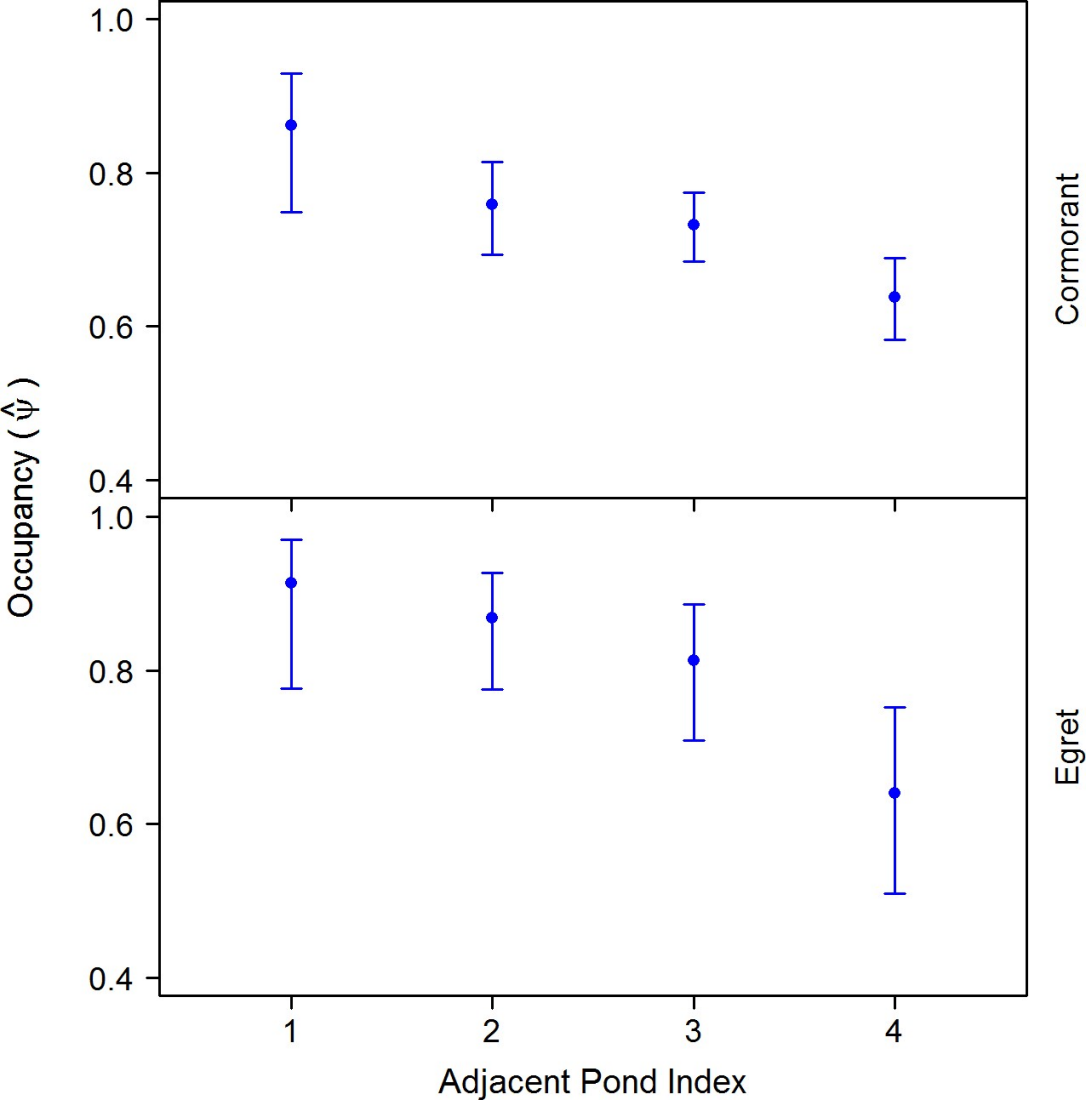
Occupancy probability (+95% CI) of cormorants and egrets on catfish ponds in the Mississippi Delta by adjacent pond index. Estimates were made using the top ranked model for each species, while keeping all other variables at their mean value. This index is based on the general isolation of catfish ponds relative to adjacent ponds (i.e. 1 = a pond with only one other adjacent pond present at any given edge).

Distance to nearest roads showed no meaningful influence on either cormorant or heron occupancy (Table 3 and Fig 3). Distance to nearest activity centers was positively related to cormorant and heron occupancy, and opposite effects were observed for distance to trees, where cormorants displayed a positive relationship and egrets a negative (Table 3 and Fig 3). Occupancy probability based on adjacent pond index for both cormorants and egrets showed a general negative trend (Table 3), where larger values of adjacent pond index had lower probabilities compared to lower adjacent index values (Fig 5)

### Resource selection function

We collected conditional pond data using onsite ground surveys on twenty three unique clusters, which resulted in 858 ponds for cormorant analysis, 384 for egrets, and 505 for herons (S2 Dataset). Oxygen level was dropped from the analysis due to low variation. In fact, only three ponds had low oxygen reported within our data for egrets, two ponds for cormorants, and none for herons. There was no evidence of collinearity among the predictor variables (VIFs < 5), we therefore constructed a model for each species with all variables.

**Fig 6:**
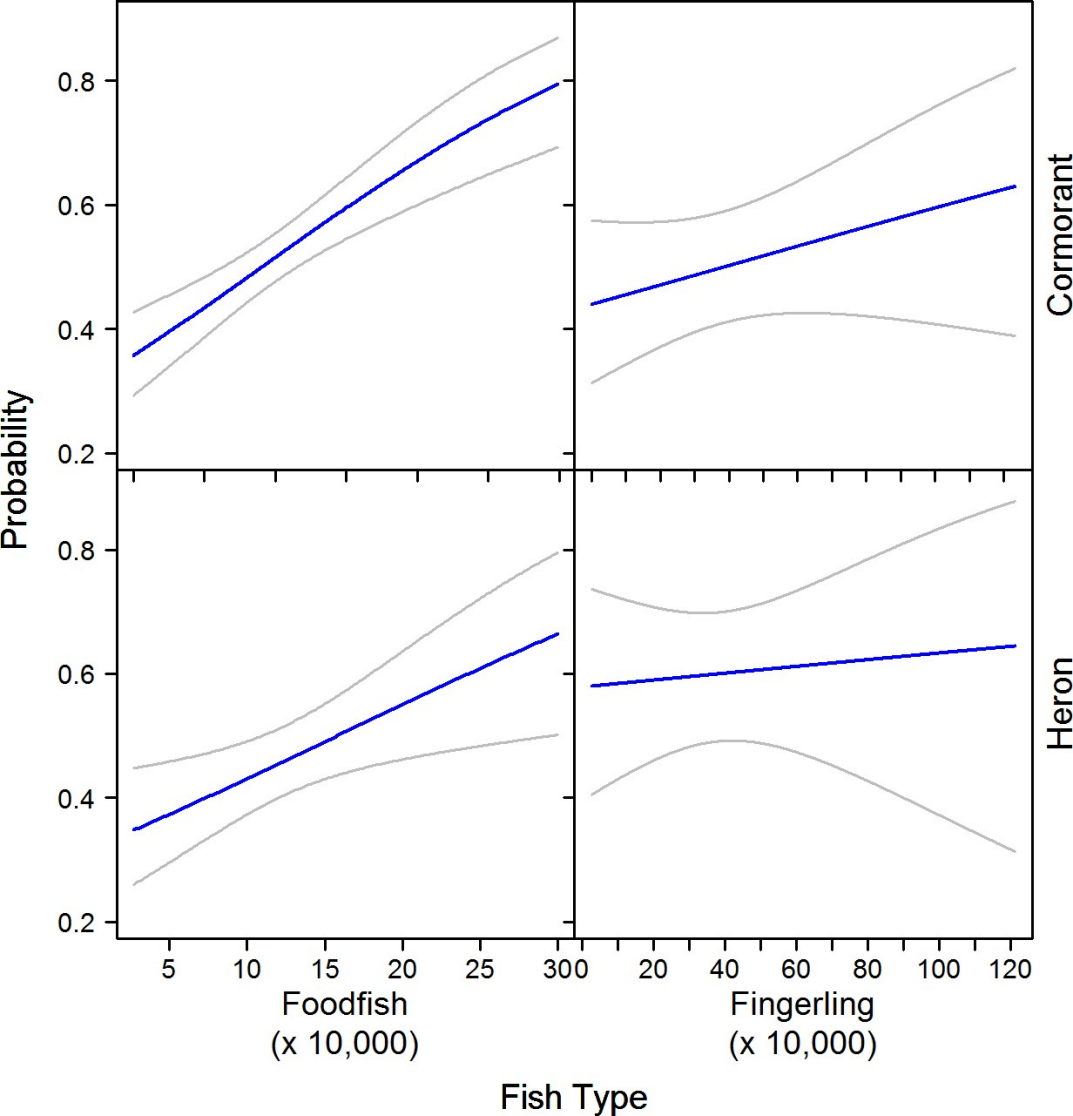
Interaction effect of fish type and fish abundance on the probability (+95% CI) of cormorants and herons use of catfish ponds in the Mississippi Delta. Estimates were made using logistic regression models of bird presence and absence on catfish ponds while holding all other variables at their mean.

The variable of disease was the only significant predictor (*p* = 0.014) of egret presence, showing ponds with disease having a higher probability of use (Table 6). We found a similar trend with heron presence and disease (*p* = 0.086). Fish abundance ranged from 15 to 430 thousand for fingerlings, and 1 to 71 thousand for foodfish. The interaction of fish type and fish abundance was significantly related to both heron and cormorant presence (Table 6). For both species, the probability of use increased on foodfish ponds as fish abundance increased, whereas a weaker relationship was observed for fingerling ponds (Fig 6).

**Table 6:** Parameter estimates, standard error, and associated p-values for resource selection functions using logistic regression for cormorants, egrets, and herons use of catfish ponds in the Mississippi Delta. Presence data were collected by flying aerial surveys of catfish ponds, and variable data were collected on ponds randomly selected with and without the target species.

	Cormorant N = 858			Egret N = 384			Heron N = 505		
Variable ^a^	β	SE	*p*	β	SE	*p*	β	SE	*p*
Intercept	-0.050			-0.104			0.101		
Disease (present)	-0.292	0.553	0.597	1.907	0.776	0.014	1.142	0.666	0.086
Other fish (present)	-0.120	0.173	0.487	0.100	0.301	0.738	0.380	0.244	0.119
Fish type (food)	0.706	0.266	0.008	0.036	0.453	0.936	-0.165	0.350	0.635
F-Abun	0.128	0.111	0.250	-0.058	0.132	0.657	0.045	0.157	0.774
Stock days	-0.060	0.070	0.393	-0.062	0.110	0.573	0.036	0.096	0.704
F-Abun × fish type	1.198	0.291	<0.001	0.382	0.337	0.257	0.895	0.396	0.023

^a^ Variable Descriptions: Disease: the presence of disease, where absent is set at the reference group; Other fish: the presence of other non-catfish fish species also in the pond, where no other fish is set as the reference group; Fish type: foodfish or fingerling, where fingerling is set as the reference group; F-Abund: total abundance of fish within a pond; Stock days: the number of days since the pond was last stocked with catfish

## Discussion

Understanding how habitat and landscape characteristics associated with catfish ponds influence piscivorous avian use provide valuable information on where depredation may be more likely to occur and the underlying environmental conditions why. Our results estimated use by three piscivorous bird species of catfish ponds within three overarching categories: 1) pond level, 2) surrounding habitat, and 3) conditional. As expected, results for each species differed in numerous ways, highlighting inherent difference in their foraging ecology. While this studies focus was in the Mississippi Delta, we believe our findings could be applicable to other aquaculture industries that report similar depredation issues. For example, Arkansas has the largest production of baitfish aquaculture and reports depredation by wading birds and cormorants [9,55]. Likewise, eastern Mississippi and Alabama culture species including catfish, tilapia, and trout, all of which experience conflict with avian predators [1,9,56]. Wading bird populations in parts of Louisiana have even been shown to relate directly with the areas crayfish aquaculture industry [57]. The foraging decision behavior of the avian species studied here are likely similar on these other production ponds.

Occupancy analysis proves to be a valuable technique when surveying species at designated sites, especially with imperfect detection [34,35]. Here, we adapted occupancy modeling in a non-traditional approach. For example, survey sites are typically larger in scale and designated based on species ecology, such as habitat type or home range [39]. Our designation of survey site was finer in scale, based on individual catfish ponds. In this framework we assumed pond use was consistent throughout the winter season. Detection of each species varied throughout each season and was related to the relative abundance of each species in the area. Thus, we were more likely to observe a target species on a used pond when there was a greater abundance of conspecifics in the region [46]. It is important to note that our detection probabilities were specific to our survey methodology. For example, detection probabilities would most likely change if we observed each pond for a longer time period or more frequently. Without the incorporation of imperfect detection, our naïve estimates of occupancy probabilities would be underestimated [50].

Pond size was positively related to occupancy of cormorants, herons, and egrets. Smaller ponds have reduced perimeters, restricting shoreline use by wading birds such as herons and egrets. Similarly, cormorants foraging in open water have limited space on small ponds and may lack the required space to take flight [36]. The effect of pond category varied among avian species. Cormorants and herons showed lesser occupancy of treatment ponds, broodfish ponds, and split-ponds stocked with foodfish. Treatment ponds do not contain fish and broodfish ponds contain larger adult catfish that would be difficult or impossible to consume [20,26]. The reduced use of split-ponds was most likely related to pond size, as split-ponds were on average 1.44 ha (SD = 0.43) and levee ponds averaged 3.67 ha (SD = 1.64). Conversely, egrets showed high occupancy of treatment, brood, and split-ponds relative to other categories. Within aquaculture areas, egret diets have been shown to consist of smaller amounts of catfish compared to cormorants and herons [4,9]. In fact, egrets have a more generalist diet and may be using brood ponds or treatment ponds as loafing areas or consuming other prey items such as invertebrates, amphibians, or reptiles [38]. For each of the study species, channel foodfish had greater occupancy probabilities compared to hybrid foodfish, although the distinction was less evident for cormorants (Fig 4). Because hybrids grow faster compared to channels they may be less sought out compared to the smaller channel catfish of similar age [28]. Similar to other studies [5], we also found herons and egrets were more likely to use diseased ponds. These wading birds hunt prey by slowly wading or standing still in wait of prey in shallow water [37,38], and sick or dead catfish are likely an easy target when hunting. In our study, 81% of ponds with disease were channel catfish. This may also explain greater use of channel fingerlings by egrets and herons. Cormorants, however, showed greater use of hybrid fingerlings compared to channel fingerling. One possible explanation is hybrid fingerlings were stocked in greater abundance on average compared to channel fingerlings. The interaction of fish type with fish abundance was stronger for cormorants and fingerlings compared to herons (Fig 6).

Cormorant and heron use of foodfish ponds was also related to fish abundance (Fig 6). Past studies that have stocked ponds with differing fish densities have found cormorants to use areas with greater densities more often [30]. In these studies, area of open water was similar, making density and total abundance equivalent. However, two ponds differing significantly in size but stocked at similar densities will result in drastic differences in fish abundance, and is why we elected to use fish abundance. Typically, foodfish ponds are stocked with catfish within the primary size class consumed by both cormorants and herons [36,37,58]. The greater abundance of available foodfish may potentially increase the capture success or encounter rate of both species. Egrets did not have a significant influence of fish abundance and fish type in our analysis. While cormorants generally consume healthy catfish [4,59], egrets consume diseased fish more readily, and therefore the abundance of fish may not be as important as the presence of moribund fish.

Distance from pond to activity center was positively related to both cormorant and egret occupancy. This finding is intuitive given these activity centers are continuously accompanied by human and vehicle presence. Cormorants showed a positive relationship with distance to nearest forested areas, meaning they are more likely to use ponds farther away from such areas. Conversely, egrets showed a negative relationship. We hypothesize nearby forest areas may limit cormorants’ ability to flee due to the distance required to take off from water and gain sufficient height to clear nearby treetops [36]. For example, Canada geese (*Branta Canadensis*) avoid areas where their flight angle is greater than 130 degrees [60]. Conversely, egrets may perceive forested areas as potential loafing or perching sites. The variable adjacent pond index was created to be a general metric of how isolated a pond was relative to adjacent ponds. Ponds with a category four were within the interior of ponds clusters, whereas a three or two represented ponds on the periphery of a cluster. Both cormorants and egrets showed higher occupancy of ponds with lower adjacent pond index numbers, indicating preference of ponds that were more isolated. Ponds on the periphery typically do not have levee roads on all sides compared to internal ponds, or the roads are less maintained, making harassment from vehicles more difficult. Such harassment is a primary scare tactic used by producers [8,61]. Similarly, ponds within the interior of clusters are generally closer to farm roads travelled more routinely either for feeding purposes or general movement to and from different parts of the facility. Such continued vehicle presence alone can elicit an effect on cormorant presence [62].

We expected to observe differences in use probabilities based on the presence of other, non-catfish species in a pond. There is evidence that cormorants [31,59], egrets, and herons [4] consume other species such as shad or *Lepomis* spp. found in catfish ponds. However, we take caution in our findings due to the nature of our survey methods. We selected clusters to collect conditional data based on species presence, and within those clusters we randomly selected ponds that did not contain the target species to also collect data from for comparison. In most cases, clusters either contained other fish species in nearly all ponds, or no other fish in nearly all ponds. Therefore, our presence/absence data contained little variation related to other fish presence.

The number of days since a pond was last stocked was not significant in any resource selection models. A past study found cormorants to have greater odds of using a pond with fewer days since last stocking [20]. However, the mean stock date during this past study was approximately ten months, whereas in our study it was approximately six months. This reduced time may be indicative of changing culture practices observed today, and may be related to the now more popular hybrid species. Regardless, our shorter stock days may be why we did not observe the same trend as the other study [20]. Presumably catfish will be larger when a greater amount of time has passed since a stocking event. It is possible the shorter amount of time since last stocking observed in our study was not sufficient enough for catfish to grow to a length less desirable to avian predators.

**Fig 7:**
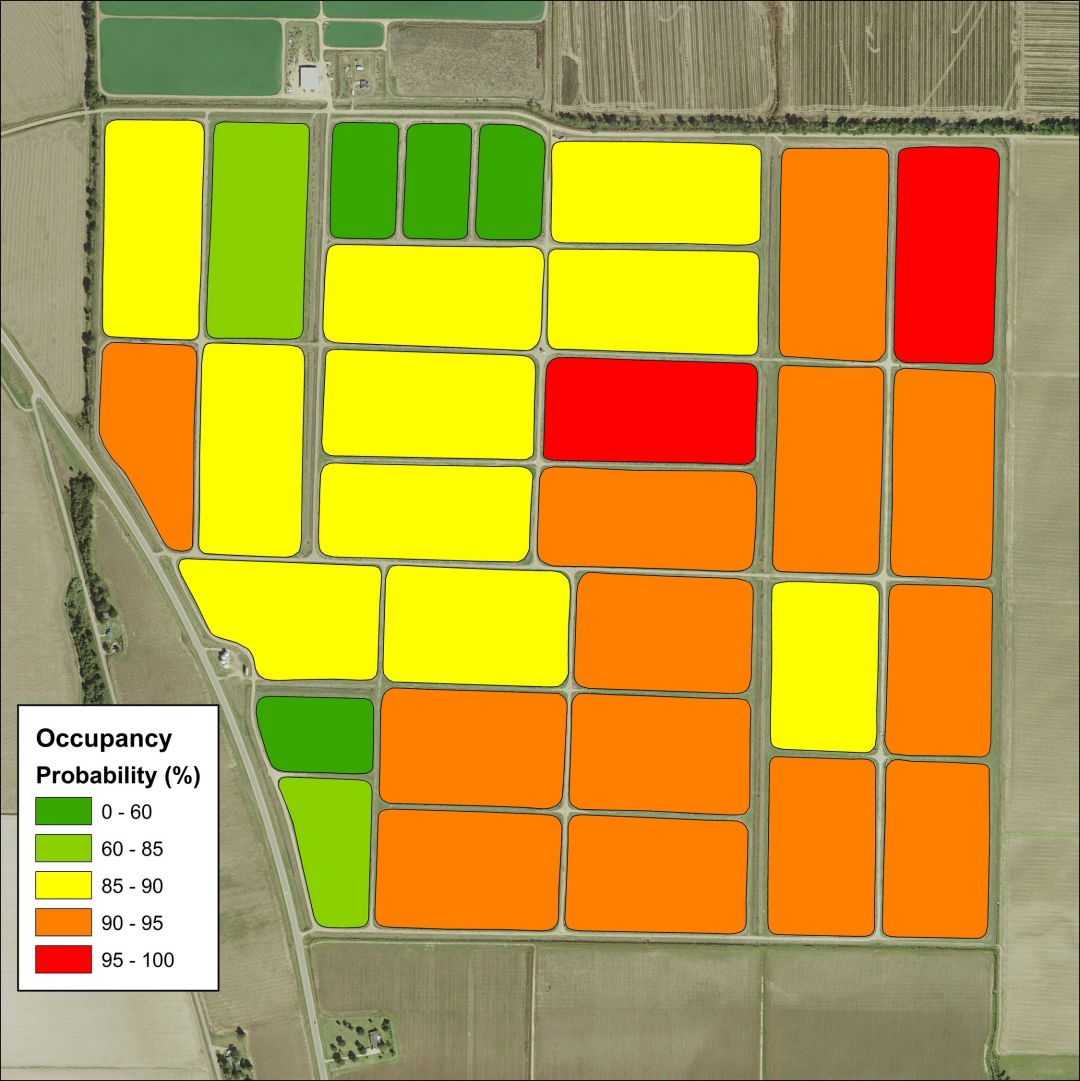
Occupancy probability of cormorants on a selected group of catfish ponds within the Mississippi Delta. Estimates were obtained by incorporating actual data of each pond into an occupancy model (Table 5). Information such as this has the potential to increase bird harassment efficiency and to inform future stocking decisions.

Our results indicate species-specific occupancy models and resource selection functions may be used to predict the probability of pond use by these avian predators during a winter season. Combining these models with GIS can produce a heat map of probabilities of catfish farms, which can be easily interpreted by producers and personnel. This information can be used to target harassment efforts with the objective to increase harassment efficiency. Using our occupancy model, we created an example of such a map for cormorant occupancy on a cluster of ponds surveyed within our sample frame (Fig 7). This map was created by incorporating data for all variables in the model. However, it is also possible to create such a map by leaving out variables, or manipulating variables to inform future stocking decisions to minimize depredation potential of more expensive fish products. Likewise, such methods could be used to inform the planning and construction of new aquaculture facilities.

## Supporting information

S1 Dataset Data and metadata used in the occupancy analyses of cormorants, herons, and egrets on catfish ponds. (XLSX)

S2 Dataset Data and metadata used in the resource selection functions of cormorants, herons, and egrets on catfish ponds. (XLSX)

## References

[1] Vilsack T, Reilly JT. 2012 Census of Aquaculture. United States Dep Agric 2014.

[2] National Agriculture Statistics Service (NASS). Catfish Production. United States Dep Agric 2018; 1–10.

[3] Vilsack T, Clark CZF. 2012 Census of Agriculture, Mississippi State and County Data. United States Dep Agric. 2014;1:685.

[4] Glahn JF, Reinhold DS, Smith P. Wading Bird Depredations on Channel Catfish Ictalurus punctatus in Northwest Mississippi. J World Aquac Soc. 1999;30: 107–114. 10.1111/j.1749-7345.1999.tb00323.x

[5] Dorr BS, Taylor JD II. Wading Bird Management and Research on North American Aquaculture Facilities. 10th Wildlife Damage Management Conference. 2003 pp. 52–61. 10.1007/s13398-014-0173-7.2

[6] Dorr BS, Burger LW, Barras SC. Evaluation of Aerial Cluster Sampling of Double-crested Cormorants on Aquaculture Ponds in Mississippi. J Wildl Manage. 2008;72: 1634–1640. 10.2193/2007-308

[7] Gorenzel WP, Conte FS, Salmon TP. Bird Damage At Aquaculture Facilities. In: Hygnstrom SE, Timm RM, Larson GE, editors. Prevention and Control of Wildlife Damage. University of Nebraska, GPAC, and USDA APHIS ADC; 1994.

[8] Wywialowski AP. Wildlife-Caused Losses for Producers of Channel Catfish Ictalurus punctatus in 1996. J World Aquac Soc. 1999;30: 461–472. 10.1111/j.1749-7345.1999.tb00994.x

[9] Glahn J, King DT. Bird Depredation In: Tucker CS, Hargreaves J, editors. Biology and Culture of Channel Catfish. Elsevier B.V.; 2004 pp. 634–657.

[10] Stickley AR, Glahn JF, King JO, King TD. Impact of Great Blue Heron Depredations on Channel Catfish Farms. J World Aquac Soc. 1995;26: 194–199. 10.1111/j.1749-7345.1995.tb00244.x

[11] Glahn J, Werner SJ, Hanson T, Engle CR. Cormorant Depredation Losses and Their Prevention At Catfish Farms: Economic Considerations USDA National Wildlife Research Center Symposia 2000 pp. 138–146.

[12] Dorr BS, Burger LW, Barras SC, Godwin KC. Economic Impact of Double-Crested Cormorant, Phalacrocorax auritus, Depredation on Channel Catfish, Ictalurus punctatus, Aquaculture in Mississippi, USA. J World Aquac Soc. 2012;43: 502–513. 10.1111/j.1749-7345.2012.00586.x

[13] US Fish and Wildlife Service (USFWS). Final Environmental Assessment. Management of Double-crested cormorant under 50 cfr 2147 and 2148. 2014.

[14] Mott DF, Boyd FL. A Review of Techniques for Preventing Cormorant Depredations at Aquaculture Facilities in the Southeastern United States. Colon Waterbirds. 1995;18: 176–180.

[15] Belant JL, Tyson LA, Mastrangelo PA. Effects of Lethal Control at Aquaculture Facilities on Populations of Piscivorous Birds. Wildl Soc Bull. 2000;28: 379–384.

[16] Blackwell B, Dolbeer R, Tyson L. Lethal Control of Piscivorous Birds at Aquaculture Facilities in the Northeast United States: Effects on Populations. N Am J Aquac. 2000;62: 300–307.

[17] Glahn J. Comparison of Pyrothechnics Versus Shooting for Dispersing Double-crested Cormorants from Their Night Roosts 19th Vertebrate Pest Conference. University of California, Davis; 2000 pp. 44–48.

[18] Johnson DH. The Comparison of Usage and Availability Measurements for Evaluating Resource Preference. Ecology. 1980;61: 65–71. 10.2307/1937156

[19] US Department of Agriculture (USDA). Catfish 2010 Part III: Changes in Catfish Health and Production Practices in the United States, 2002–09. USDA-APHIS-VS-CEAH-NAHMS Fort Collins, CO #5971111. 2010;

[20] Dorr BS. Distribution, Abundance, and Economic Impacts of Double-crested Cormorants on Channel Catfish Aquaculture in the Yazoo Basin of Mississippi Dissertation, Mississippi State University 2006.

[21] US Department of Agriculture (USDA). Catfish 2003 Part I: Reference of Fingerling Catfish Health and Production Practices in the United States, 2003. USDA-APHIS-VS-CEAH-NAHMS, Fort Collins, Co #N4061103. 2003;

[22] Tucker CS, Kingsbury SK. High-density Split-pond Systems Offer High Output, Low Maintenance. Glob Aquac Advocate. 2010; 64–65.

[23] US Department of Agriculture (USDA). Reference of Catfish Health and Production Practices in the United States, 2009. USDA-APHIS-VS, CEAH Fort Collins, CO #5801210. 2010;

[24] Glahn JF, Dorr BS, Harrel JB, Khoo L. Foraging Ecology and Depredation Management of Great Blue Herons at Mississippi Catfish Farms. J Wildl Manage. 2002;66: 194–201.

[25] Glahn JF, Dixson PJ, Littauer GA, McCoy RB. Food Habits of Double-crested Cormorants Wintering in the Delta Region of Mississippi. Colon Waterbirds. 1995;18: 158–167.

[26] Glahn J, Dorr BS, Tobin ME. Captive Great Blue Heron Predation on Farmed Channel Catfish Fingerlings. N Am J Aquac. 2000;62: 149–156.

[27] Johnson K, Engle C, Wagner B. Comparative Economics of US Catfish Production Strategies: Evidence from a Cross-sectional Survey. J World Aquac Soc. 2014;45: 279–289. 10.1111/jwas.12117

[28] Li MH, Robinson EH, Manning BB, Yant DR, Chatakondi NG, Bosworth BG, et al Comparison of the Channel Catfish, Ictalurus punctatus (NWAC103 Strain) and the Channel × Blue Catfish, I. punctatus × I.furcatus, F1 Hybrid for Growth, Feed Efficiency, Processing Yield, and Body Composition. J Appl Aquac. 2004;15: 1045–4438. 10.1300/J028v15n03

[29] Dunham R, Masser M. Production of Hybrid Catfish. Southern Regional Aquaculture Center Publication No 190 2012 pp. 1–8.

[30] Werner SJ, Dorr BS. Influence of Fish Stocking Density on the Foraging Behavior of Double-crested Cormorants, Phalacrocorax auritus. J World Aquac Soc. 2006;37: 121–125. 10.1111/j.1749-7345.2006.00015.x

[31] Stickley AR, Warrick GL, Glahn JF. Impact of Double-crested Cormorant Depredations on Channel Catfish Farms. J World Aquac Soc. 1992;23: 192–198.

[32] Glahn JF, Brugger KE. The Impact of Double-crested Cormorant on the Mississippi Delta Catfish Industry: a Bioenergetics Model. Colon Waterbirds. 1995;18: 168–175.

[33] Wires LR, Cuthbert FJ, Trexel DR, Joshi AR. Status of the Double-crested Cormorant (Phalacrocorax auritus) in North America. Final Rep to USFWS. 2001;

[34] Mackenzie DI, Nichols JD, Royle JA, Pollock KH, Bailey LL, Hines JE. Occupancy Estimation and Modeling: Inferring Patterns and Dynamics of Species Occurrence. Acedemic Press: New York; 2006.

[35] Bailey LL, Mackenzie DI, Nichols JD. Advances and Applications of Occupancy Models. Methods Ecol Evol. 2014;5: 1269–1279. 10.1111/2041-210X.12100

[36] Dorr BS, Hatch JJ, Weseloh DVC. Double-crested Cormorant (Phalacrocorax auritus), The Birds of North America Online (A. Poole. Ed.). Ithaca: Cornell Lab of Ornithology; Retrieved from the Birds of North America Online. 2014.

[37] Vennesland RG, Butler RW. Great Blue Heron (Ardea herodias), The Birds of North America Online (A. Poole. Ed.). Ithaca: Cornell Lab of Ornithology; Retrieved from the Birds of North America Online. 2011.

[38] Mccrimmon D, Ogden JC, Bancroft GT. Great Egret (Ardea alba), The Birds of North America Online (A. Poole. Ed.). Ithaca: Cornell Lab of Ornithology; Retrieved from the Birds of North America Online. 2011.

[39] Linden DW, Fuller AK, Royle JA, Hare MP. Examining the Occupancy-density Relationship for a Low-density Carnivore. J Appl Ecol. 2017;54: 2043–2052. 10.1111/1365-2664.12883

[40] Fuller AK, Linden DW, Royle JA. Management Decision Making for Fisher Populations Informed by Occupancy Modeling. J Wildl Manage. 2016;80: 794–802. 10.1002/jwmg.21077

[41] Crum NJ, Fuller AK, Sutherland CS, Cooch EG, Hurst J. Estimating Occupancy Probability of Moose Using Hunter Survey Data. J Wildl Manage. 2017;81: 521–534. 10.1002/jwmg.21207

[42] Fiske IJ, Chandler RB. Unmarked: An R Package for Fitting Hierarchical Models of Wildlife Occurrence and Abundance. J Stat Softw. 2011;43: 1–23. 10.18637/jss.v043.i10

[43] R Core Team. R: A Language and Environment for Statistical Computing. R Foundation for Statistical Computing, Vienna, Austria 2018 Available: https://www.r-project.org/

[44] Peterman WE, Crawford JA, Kuhns AR. Using Species Distribution and Occupancy Modeling to Guide Survey Efforts and Assess Species Status. J Nat Conserv. 2013;21: 114–121. 10.1016/j.jnc.2012.11.005

[45] Bailey LL, Simons TR, Pollock KH. Estimating Site Occupancy and Species Detection Probability Parameters for Terrestrial Salamanders. Ecol Appl. 2004;14: 692–702.

[46] Royle JA, Nichols JD. Estimating Abundance from Repeated Presence-Absence Data or Point Counts. Ecology. 2003;84: 777–790.

[47] Schielzeth H. Simple Means to Improve the Interpretability of Regression Coefficients. Methods Ecol Evol. 2010;1: 103–113. 10.1111/j.2041-210X.2010.00012.x

[48] Zuur AF, Ieno EN, Walker NJ, Saveliev AA, Smith GM. Mixed Effects Models and Extensions in Ecology with R. Springer Science & Business Media; 2009.

[49] Burnham KP, Anderson DR. Model Selection and Multimodel Inference: a Practical Information-theoretic Approach. Second Edi. Springer: New York; 2002.

[50] MacKenzie DI, Bailey LL. Assessing the Fit of Site-Occupancy Models. J Agric Biol Environ Stat. 2004;9: 300–318. 10.1198/108571104X3361

[51] Mazerolle MJ. AICcmodavg: Model Selection and Multimodel Inference based on (Q)AIC(c). R package version 21–1. 2017. Available: https://cran.r-project.org/package=AICcmodavg

[52] Bates D, Maechler M, Bolker B, Walker S. Fitting Linear Mixed-Effects Models Using lme4. J Stat Softw. 2015;67: 1–48. 10.18637/jss.v067.i01

[53] Manly BFJ, McDonald LL, Thomas DL, McDonald TL, Erickson WP. Resource Selection by Animals: Statistical Design and Analysis for Field Studies. 2nd ed. Kluwer Academic Publishers; 2002.

[54] US Department of Agriculture (USDA). Catfish 2010 Part II: Health and Production Practices for Foodsize Catfish in the United States, 2009. USDA-APHIS-VS, CEAH Fort Collins, CO #5950611. 2010.

[55] Werner SJ, Harrel JB, Wooten DE. Foraging Behavior and Monetary Impact of Wading Birds at Arkansas Baitfish Farms. J World Aquac Soc. 2005;36: 354–362.

[56] Dorr BS, King DT, Tobin ME, Harrel JB, Smith PL. Double-crested Cormorant Movements in Relation to Aquaculture in Eastern Mississippi and Western Alabama. Waterbirds. 2004;27: 147–154.

[57] Fleury BE, Sherry TW. Long-term Population Trends of Colonial Wading Birds in the Southern United States: the Impact of Crayfish Aquaculture on Louisiana Populations. Auk. 1995;112: 613–632.

[58] Tucker C, Hargreaves J. Biology and Culture of Channel Catfish. Elsevier B.V.; 2004.

[59] Glahn J, Dorr BS. Captive Double-crested Cormorant Phalacrocorax auritus Predation on Channel Catfish Ictalurus punctutus Fingerlings and Its Influence on Single-batch Cropping Production. J World Aquac Soc. 2002;33: 85–93.

[60] Gosser AL, Conover MR, Messmer TA. Managing Problems Caused by Urban Canada Geese Berryman Institute Publication 13. Utah State University, Logan; 1997.

[61] Reinhold DS, Sloan CA. Strategies To Reduce Double-Crested Cormorant Depredation at Aquaculture Facilities in Mississippi Symposium on double-crested cormorants: population status and management issues in the Midwest, USDA National Wildlife Research Center Symposia 1997 pp. 99–105.

[62] Conover M. Resolving Human-Wildlife Conflict: The Science of Wildlife Damage Management. Lewis Publication, Washington D.C; 2000.

